# Genome Sequence of a Staphylococcus xylosus Clinical Isolate, Strain SMA0341-04 (UGA5), from Siaya County Referral Hospital in Siaya, Kenya

**DOI:** 10.1128/MRA.01625-18

**Published:** 2019-04-18

**Authors:** Qiuying Cheng, Gary Xie, Hajnalka Daligault, Karen Davenport, Cheryl Gleasner, Lindsey Jacobs, Jessica Kubicek-Sutherland, Tessa LeCuyer, Vincent Otieno, Evans Raballah, Norman Doggett, Benjamin McMahon, Douglas J. Perkins, Harshini Mukundan

**Affiliations:** a Center for Global Health, Department of Internal Medicine, University of New Mexico Health Sciences Center, Albuquerque, New Mexico, USA; b Biosecurity and Public Health, Bioscience Division, Los Alamos National Laboratory, Los Alamos, New Mexico, USA; c Theoretical Biology and Biophysics, Theoretical Division, Los Alamos National Laboratory, Los Alamos, New Mexico, USA; d Physical Chemistry and Applied Spectroscopy, Chemistry Division, Los Alamos National Laboratory, Los Alamos, New Mexico, USA; e University of New Mexico Laboratories of Parasitic and Viral Diseases, Kisumu, Kenya; f Department of Medical Laboratory Sciences, School of Public Health, Biomedical Sciences and Technology, Masinde Muliro University of Science and Technology, Kakamega, Kenya; University of Maryland School of Medicine

## Abstract

We report here the genome sequence of a Staphylococcus xylosus clinical isolate, strain SMA0341-04 (UGA5), which contains one chromosome and at least one plasmid. Notably, strain SMA0341-04 (UGA5) contains the tetracycline efflux major facilitator superfamily (MFS) transporter (*tetK*) gene.

## ANNOUNCEMENT

Staphylococcus xylosus, a commensal Gram-positive bacterium present on the skin of humans and animals (1, 2), is a ubiquitous bacterium that is naturally present in a wide range of foodstuffs (3, 4). *S. xylosus* has traditionally been regarded as an apathogenic member of the coagulase-negative staphylococci (CoNS) and is utilized for many applications, including fermentation of meat and dairy products (5). However, some virulent strains may participate in opportunistic infections in humans and other mammals (6–10). We report here the draft genome sequence of *S. xylosus* strain SMA0341-04 (UGA5), isolated from the venous blood of a febrile female pediatric patient (6.1 months old) at the Siaya County Referral Hospital in western Kenya in 2004. Diagnostic results revealed that the bacteremic patient was HIV negative but had Plasmodium falciparum malaria.

Prior to any treatment interventions, upon admission, blood was collected into a pediatric isolator 1.5 microbial tube (Wampole Laboratories, Cranbury, NJ) and cultivated at 35°C for 18 to 24 hours in 5% CO_2_ on 5% sheep blood agar. Bacterial DNA was extracted from a pure culture using the UltraClean microbial DNA isolation kit (Qiagen, Germantown, MD) according to the manufacturer’s instructions with minimal modifications. The library was prepared from 100 ng of bacterial DNA using the NEBNext Ultra DNA library prep kit for Illumina (New England Biolabs, Ipswich, MA). *S. xylosus* SMA0341-04 (UGA5) was draft sequenced to 529-fold coverage using a MiSeq version 2 500-cycle sequencing kit (Illumina, San Diego, CA), resulting in 18,934,198 paired-end 251-bp reads. BWA version 0.7.2 (11) was used to map 99.74% of the SMA0341-04 (UGA5) reads onto UGA contigs, and mapped SMA0341-04 (UGA5) reads covered 77.4% of the *S. xylosus* strain HKUOPL8 chromosome. The data quality was assessed, and the data files were filtered and trimmed with FaQCs version 1.3 (12) and then assembled with Velvet version 1.2.08 (13, 14) and with IDBA version 1.1.0 (15). The consensus sequences were computationally shredded and reassembled with Phrap version SPS-4.24 (16, 17) to allow some manual editing with Consed (18), resulting in a final 16 (99.74% of the reads) contigs of >500 bp with an *N*_50_ value of 904,770 bp for SMA0341-04 (UGA5). The draft genome of SMA0341-04 (UGA5) consists of a 2,866,950-bp sequence with an average G+C content of 32.8%. Annotations were completed at the Los Alamos National Lab (LANL) using an automated system using the Ergatis workflow manager v.2.0 (19) and in-house scripts.

There are 2,715 predicted protein coding genes, 53 tRNA genes, and 6 rRNA genes within the genome of SMA0341-04 (UGA5). Among them, 38% of the protein coding genes were annotated in a SEED subsystem (20), whereas 62% were not associated with a SEED subsystem. A total of 748 genes were annotated as hypothetical proteins. Of all the predicted genes, 2,437 are common between SMA0341-04 (UGA5) and the *S. xylosus* strain HKUOPL8 genomes, with 278 genes being unique to SMA0341-04 (UGA5). A total of 21 genes in the subsystem are associated with resistance to antibiotics. Similar to other methicillin-susceptible CoNS strains, SMA0341-04 (UGA5) is highly sensitive to various antimicrobial drugs (21), except tetracycline, for which resistance was detected using the disk diffusion method according to the Clinical and Laboratory Standards Institute guidelines (22). This is consistent with the presence of the tetracycline efflux major facilitator superfamily (MFS) transporter encoded by the *tetK* gene on the rep7 type plasmid of SMA0341-04 (UGA5).

### Data availability.

The GenBank accession number for Staphylococcus xylosus SMA0341-04 (UGA5) is NWQI00000000, the BioProject accession number is PRJNA407859, the BioSample accession number is SAMN07665175, and the SRA accession number is SRR8649745.
